# Identification of differential expressed lncRNAs in human thyroid cancer by a genome‐wide analyses

**DOI:** 10.1002/cam4.1627

**Published:** 2018-06-20

**Authors:** Wei Lu, Yongcan Xu, Jiewei Xu, Zhong Wang, Guochao Ye

**Affiliations:** ^1^ Department of General Surgery Huzhou Central Hospital Huzhou China

**Keywords:** biomarker, LINC00704, lncRNAs, profiling, thyroid cancer

## Abstract

Recently, a growing number of evidence has revealed that long noncoding RNAs (lncRNAs) act as key regulators in various cellular biologic processes, and dysregulation of lncRNAs involves in tumorigenesis and cancer progression. However, the expression pattern, clinical relevance, and biologic function of most lncRNAs in human thyroid cancer remain unclear. To identify more thyroid‐cancer‐associated lncRNAs, we analyzed the expression profile of lncRNAs in thyroid cancer tissues and adjacent normal or non‐tumor tissues using RNA sequencing data and gene microarray data from The Cancer Genome Atlas and Gene Expression Omnibus. Annotation and analyses of these data revealed that hundreds of lncRNAs are differentially expressed in thyroid cancer tissues when compared with normal tissues. By copy number variation analyses, we identified that some of those dysregulated lncRNAs genome locus are accompanied with the copy number amplification or deletion. Moreover, some lncRNAs expression levels are significantly associated with thyroid cancer patients overall or recurrence‐free survival time, such as RUNDC3A‐AS1, FOXD2‐AS1, PAX8‐AS1, and CRYM‐AS1. Furthermore, we validated an lncRNA termed LINC00704 in thyroid cancer cells by performing loss of function assays. Downregulation of LINC00704 could significantly impair thyroid cancer cells proliferation, colony formation, inhibit cell‐cycle progression and cell invasion, and induce cell apoptosis. Taken together, our findings reveal that lots of lncRNAs are dysregulated and may play critical roles in thyroid cancer, and this study could provide useful resource for identification and investigation of novel lncRNA candidates for thyroid cancer.

## 1 INTRODUCTION

Thyroid cancer is the most common type of malignancy in endocrine, which stems from parafollicular or follicular thyroid cells.^1^ Several histologic types of thyroid cancer have been identified, including follicular thyroid carcinoma, papillary thyroid carcinoma, and anaplastic thyroid carcinoma.^2^ Although the advance on surgery, chemotherapy, and radiation therapy has led to a favorable survival rate of thyroid cancer patients, the incidence of thyroid cancer has significantly increased worldwide over the past few decades.^3^, ^4^ Although genetic abnormalities and environmental factors have been found to be involved in thyroid carcinogenesis,^5^ the molecular mechanisms of thyroid carcinoma pathogenesis remain unclear. Therefore, it is urgent to better understand the molecular mechanisms of initiation and progression of thyroid cancer, which may benefit the diagnosis and treatment of thyroid cancer.

Over the past decade, findings from the ENCODE (Encyclopedia of DNA Elements) consortium have revealed that a great number of noncoding transcripts are transcribed from human genome.^6^ Interestingly, numerous noncoding RNAs including microRNAs, long noncoding RNAs (lncRNAs), and pseudogenes have been discovered after annotation of these noncoding transcripts.^7^, ^8^ lncRNAs are a new member of ncRNA family, which is longer than 200 nt and lacking of protein coding capacity.^9^ Recent studies have revealed that lncRNAs are widely expressed in many human tissues and cells, and participate in many important cellular process, such as X chromatin imprinting, stem cells differentiation, immune response, tumor cells growth, and chemotherapy resistance.^10^, ^11^ Dysregulation of lncRNAs expression has been found to involve in the development of various human diseases.^12^ Moreover, lncRNA and gene profiling analyses in a large cohort of multiple types of cancer tissues by high‐throughput RNA sequencing or microarray uncovered that thousands of lncRNAs are differentially expressed in human cancer tissues.^13^ Meanwhile, a growing number of studies have validated a lot of lncRNAs function and underlying mechanisms in diverse cancers cells. For example, Sun et al found that gastric‐cancer‐associated lncRNA HOXA11‐AS is over‐expressed in gastric cancer tissues and cells, and promotes cell growth and metastasis through functioning as a competing endogenous RNA for miR‐1297 and scaffolding the PRC2, LSD1, and DNMT1.^14^ In addition, lncRNA CASC9 promotes cells metastasis through interacting with transcriptional coactivator CREB‐binding protein, thereby upregulating LAMC2 expression in Esophageal squamous cell carcinoma.^15^

In the case of thyroid cancer, a growing number of studies have illustrated the biologic function and underlying mechanism of a part of differentially expressed lncRNAs.^16^ For example, lncRNA MALAT1 expression is upregulated in thyroid cancer, and promotes cell proliferation and invasion through regulating the expression of IQGAP1.^17^ Liu et al reported that the expression of H19 is much higher in thyroid cancer tissues and cells, and over‐expressed H19 promotes cell growth, migration, and invasion via acting as a competitive endogenous RNA (ceRNA) for miR‐17‐5p and antagonizing the repression of miR‐17‐5p on its target YES1.^18^ Moreover, Dai et al discovered that AFAP1‐AS1 expression was increased in thyroid cancer tissues, and downregulation of AFAP1‐AS1 expression could inhibit cell proliferation, induce apoptosis, and decreases invasion in thyroid cancer.^19^ Although a few of lncRNAs have been characterized in thyroid cancer, the expression pattern and clinical relevance of most lncRNAs in thyroid cancer remain unclear. To identify more thyroid‐cancer‐associated lncRNAs, we performed genome‐wide analyses to differential profiling lncRNAs expression in thyroid cancer by analyzing TCGA RNA sequencing data in thyroid cancer tissues and adjacent normal samples, and three independent gene microarray data from GEO. This study reveals the differentially expressed lncRNAs in human thyroid cancer, which may provide useful candidates for thyroid cancer diagnosis and treatment.

## 2 MATERIALS AND METHODS

### 2.1 TCGA RNA sequencing data and public microarray data analyses

The TCGA thyroid cancer tissues and normal specimen RNA sequencing data and corresponding clinical data were downloaded from https://gdac.broadinstitute.org/. Other three public thyroid cancer RNA sequencing and microarray datasets (GSE83520,^20^ GSE66783,^21^ and GSE103254) were downloaded from Gene Expression Omnibus (GEO). Analyses of lncRNAs expression profile from GEO microarray datasets was based on the Agilent‐060228 Human LncRNA v5 4X180K, and Arraystar Human LncRNA microarray V2.0 platforms. These lncRNA microarray data were preprocessed by R software and packages.

### 2.2 lncRNAs genomic loci copy number variation analyses

The raw somatic gene copy number variation data of thyroid cancer tissues was downloaded from Broad GDAC FireBrowser website (https://gdac.broadinstitute.org/). Next, the GISTIC 2.0 was used to determine the significantly copy number amplifications and deletions of recurrent lncRNAs genomic regions.^22^ Then, each of the lncRNAs genomic regions was mapped to the GISTIC peaks. The copy number amplification or deletion Peaks with *q* values less than 0.25 was considered significant. Finally, the number of lncRNAs in peaks, focal/broad frequencies, and *q* values of all peaks were summarized at gene level.

### 2.3 Analyses of thyroid cancer survival associated lncRNAs

To illustrate the relationship between differentially expressed lncRNAs and thyroid cancer patients survival time and identify overall survival (OS) and recurrence‐free survival (RFS)‐associated lncRNAs, the univariable Cox PH regression analyses were conducted. Next, we used multivariable Cox PH regression analyses to evaluate each of differentially expressed lncRNAs as dependent variable factor. Then, thyroid cancer patients were divided into high‐ and low‐lncRNA expression groups according to the median expression level of each lncRNA. The lncRNA with log‐rank *P* value less than .05 between high and low expression groups was defined statistically significant. Bio‐conductor and R software was used for all these analyses.

### 2.4 Cell culture and transfection

Thyroid cancer cell line BHT101 and BCPAP was purchased from the Type Culture Collection of the Chinese Academy of Sciences (Shanghai, China). BHT101 cell was grown in DMEM medium (Gibco) containing 20% heat‐inactivated FCS (Gibco) and glutamine; BCPAP cells was maintained in RPMI 1640 medium containing 10% fetal bovine serum (Gibco) and 2 mmol/L l‐glutamine (Invitrogen, Carlsbad, CA) at 37°C with 5% CO_2_. The LINC00704 and negative control siRNAs (Invitrogen, Carlsbad, CA) were transfected into BHT101 and BCPAP cells using RNAiMAX (Invitrogen) according to the manufacturer's protocol. Forty‐eight hours after transfection, the cells were harvested for further experiment. The LINC00704 siRNA sequences are siRNA 1#, 5′‐GCUUGCUCUCACAGCCAUUTT‐3′ siRNA 2#, 5′‐GGAACCUCCUUUCUUACAUTT‐3′.

### 2.5 RNA extraction and qRT‐PCR

The total RNA of BHT101 and BCPAP cells was extracted using the RNeasy Purification Kit (QIAGEN) according to the manufacturer's manual. Then, 1 μg of total RNA was reverse transcribed into cDNA using iScript Reverse Transcription Supermix (Bio‐Rad, CA, USA). The iTaq Universal SYBR Green Supermix (Bio‐Rad) was used to detect LINC00704 expression, and the house keeping gene GAPDH was used as internal control. The primer sequence of LINC00704 is forward 5′‐TGCGTTCAGTAAAACGGGCA‐3′ and reverse 5′‐TGTGGGAAATGCAGGGTTCT‐3′. The primer sequence of GAPDH is forward 5′‐AGAAGGCTGGGGCTCATTTG‐3′ and reverse 5′‐ AGGGGCCATCCACAGTCTTC‐3′. qRT‐PCR assays were conducted on CFX96 Touch Real‐Time PCR Detection System (Bio‐Rad), and comparative cycle threshold (CT) (2^−ΔΔCT^) method was used for the data analyses.

### 2.6 Cell proliferation and colony formation assays

The BHT101 and BCPAP cells were transfected with LINC00704 or negative control siRNAs, and seeded into 96‐well plate with 3000 cells per well after transfection for 48 hours. Next, 10 μL of the CCK8 regent (Dojindo Molecular Technologies) was added into each well, and the CCK8‐treated cells were incubated for 2 or 3 hours at 37°C. Then, the OD450 absorbance value of each well was obtained every 24 hours. For colony formation assays, BHT101 and BCPAP cells transfected with LINC00704 or negative control siRNAs were plated into 6‐well plate with 2000 cells per well. The medium was changed every 4 days, and the clone was fixed by Methanol for 15 minutes, and stained by 0.5% purple crystal for 15 minutes.

### 2.7 Flow cytometric assays

For the cell apoptosis analyses, BHT101 and BCPAP cells transfected with LINC00704 or negative control siRNAs were harvested at 48 hours after transfection. The collected cells were double stained with FITC‐annexin V and propidium iodide (PI), and then analyzed on flow cytometer (FACScan; BD Biosciences) equipped with CellQuest software (BD Biosciences). For cell‐cycle analyses, the BHT101 and BCPAP cells transfected with LINC00704 or negative control siRNAs were stained with PI using the CycleTEST PLUS DNA Reagent Kit (BD Biosciences) according to the manufacturer's manual and analyzed on FACScan. The percentage of cells in G0/G1, S, and G2/M phases was calculated.

### 2.8 Transwell assays

Transwell assay (Corning, Tewksbury, MA, USA, 8.0‐μm pores) was performed to examine BHT101 and BCPAP cells invasion ability after transfection with LINC00704 or negative control siRNAs. 1 × 10^5^ cells in 350 μL medium were added into the upper chamber of insert coated with Matrigel (Sigma‐Aldrich), and 700 μL medium containing 10% FBS or 20% FCS was added to the lower chamber. Twenty‐four hours post‐incubation at 37°C, the BHT101 and BCPAP cells invaded through the membrane were fixed by methanol for 15 minutes, stained with 0.5% crystal violet for 15 minutes, and imaged using by IX71 inverted microscope (Olympus, Tokyo, Japan).

### 2.9 Western blot analysis

BHT101 and BCPAP cells were lysed by RIPA reagent (Thermo Fisher) supplemented with protease inhibitor cocktail (Roche). Next, protein in cell lysates was separated by 4%‐10% sodium dodecyl sulfate‐polyacrylamide gel and transferred to 0.22 μm PVDF membrane (Millipore). Membrane was inhibited using 5% milk and incubated with specific antibodies at 4°C overnight. The specific band was detected by incubation with ECL chromogenic substrate and quantified by densitometry (Quantity One software; Bio‐Rad, Hercules, CA, USA). GAPDH, E‐cadherin, N‐cadherin, Vimentine, and β‐catenin antibodies were purchased from Cell Signaling Technology.

### 2.10 Statistical analysis

The one‐way ANOVA and Student's *t* test (two‐tailed) were used to analyze qRT‐PCR, and in vitro CCK8 et al assays data using SPSS 20.0. *P* value < .05 was considered statistically significant.

## 3 RESULTS

### 3.1 Differential profiling of lncRNAs expression in thyroid cancer

**Figure 1:**
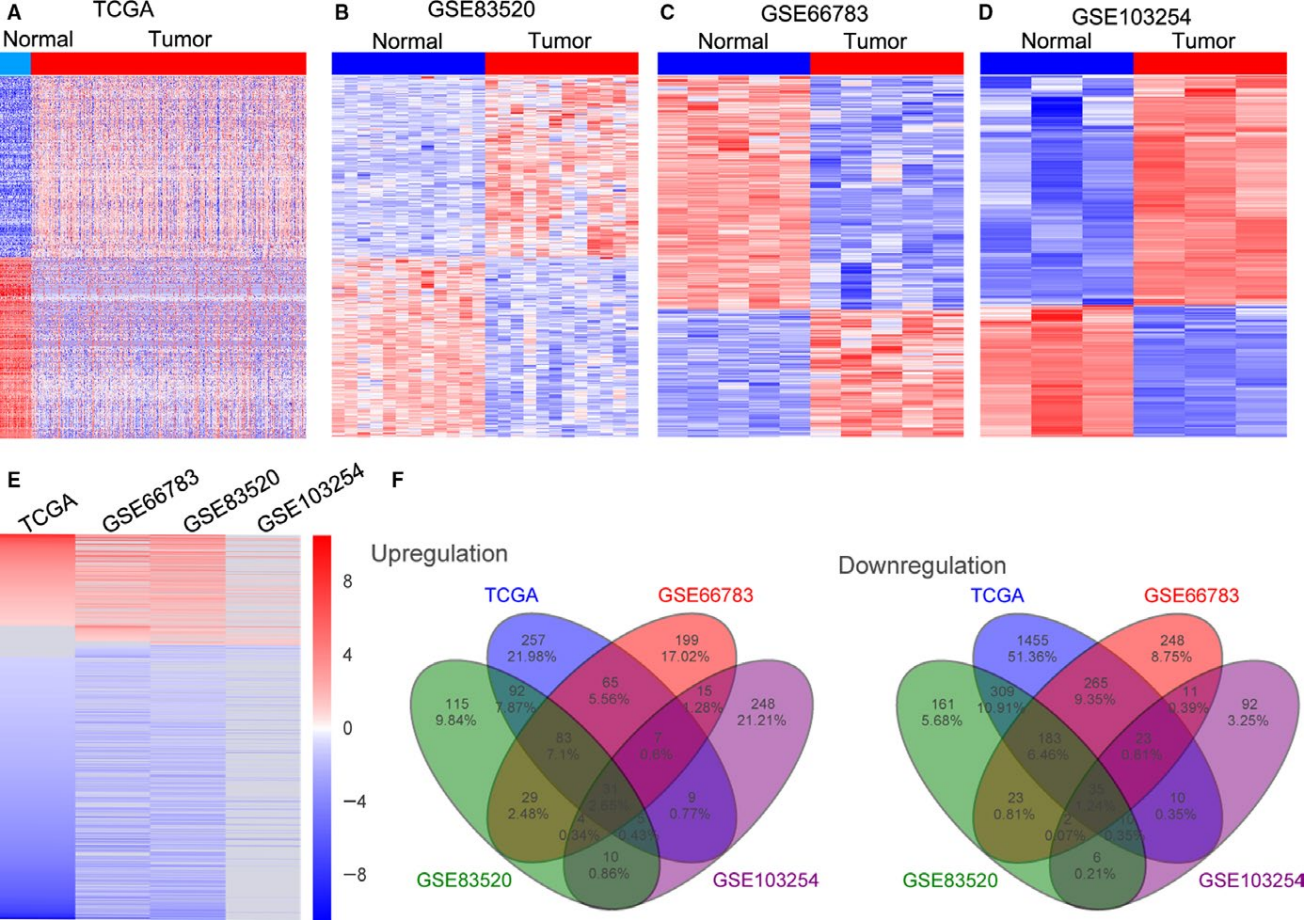
Differential profiling of lncRNAs expression in thyroid cancer tissues and normal tissues. A, A heatmap was drawn to show the dysregulated lncRNAs in thyroid cancer and normal tissue specimen using the TCGA RNA sequencing data. B‐D, Heatmaps were drawn to show the differentially expressed lncRNAs in thyroid cancer tissues compared with normal or non‐tumor tissues using the GSE83520, GSE66783, and GSE103254 datasets. E, A heatmap was drawn to show the altered lncRNAs profile (consistently altered at least two datasets, fold change) in TCGA, GSE83520, GSE66783, and GSE103254 datasets. F, Venn diagram of altered lncRNAs in TCGA, GSE83520, GSE66783, and GSE103254 datasets

To identify the differentially expressed lncRNAs in human thyroid cancer tissues, we downloaded the thyroid cancer and normal thyroid tissues RNA sequencing data from TCGA, and public thyroid cancer RNA sequencing and lncRNA microarray data from GEO (GSE83520, GSE66783, and GSE103254). The TCGA dataset consists of 505 thyroid cancer samples and 59 normal tissue samples, whereas the GSE83520 dataset includes 12 paired specimens; GSE66783 consists of five paired samples; GSE103254 consists of three paired samples. Further lncRNA differential analyses revealed that 2839 lncRNAs were dysregulated in thyroid cancer from the TCGA dataset (549 upregulated and 2290 downregulated); 742 lncRNAs were differentially expressed in the GSE83520 dataset (272 upregulated and 470 downregulated); 1223 lncRNAs were dysregulated in the GSE66783 dataset (432 upregulated and 791 downregulated); 518 lncRNAs were differentially expressed in the GSE103254 dataset (329 upregulated and 189 downregulated) (Figure 1A‐D, and Table S1). Further Venn analyses showed that 350 lncRNAs expression was consistently increased and 877 lncRNAs were decreased in at least two datasets (Figure 1E‐F). These results indicate that thousands of lncRNAs are dysregulated in thyroid cancer tissue, which may be potential useful biomarkers for thyroid cancer diagnosis.

### 3.2 Copy number variations analyses of lncRNAs loci in thyroid cancer

**Figure 2:**
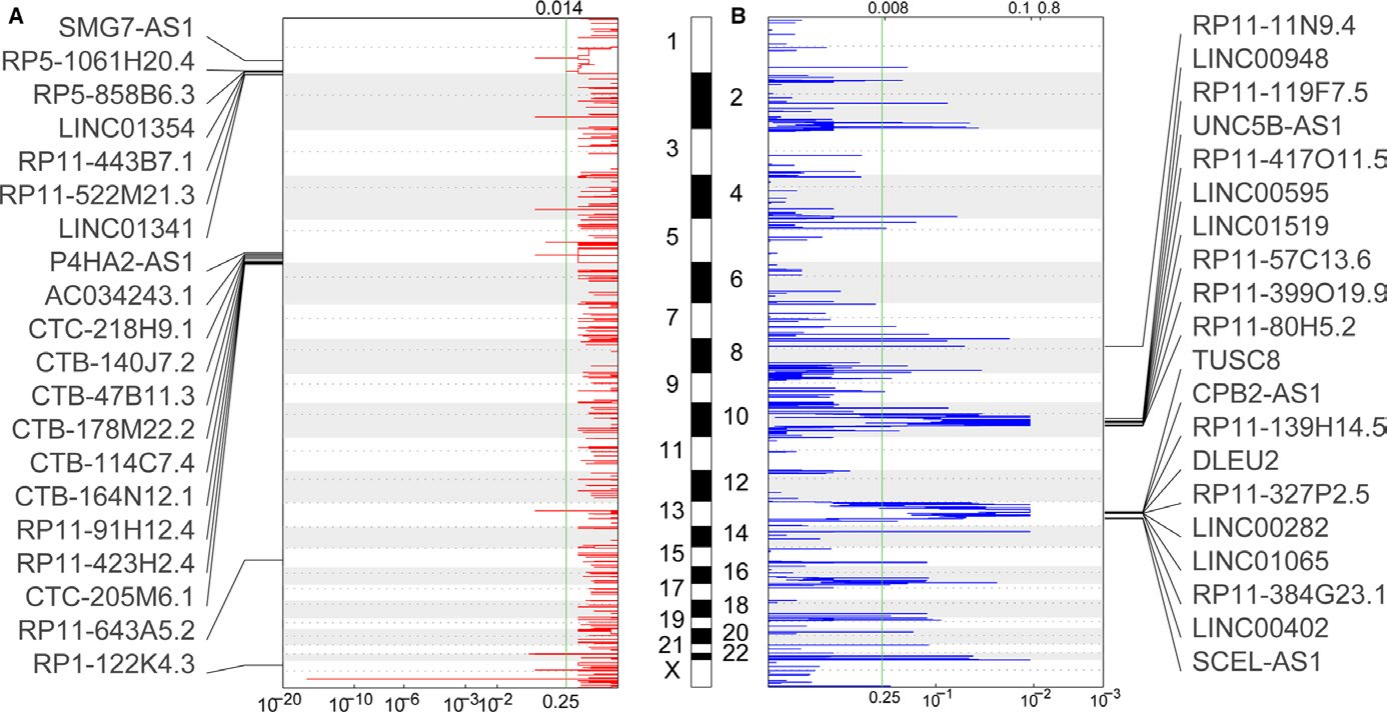
Analyses of copy number gain and loss in lncRNAs’ genome loci in thyroid cancer. A, The frequency of lncRNAs copy number gain (red) in thyroid cancer tissues was shown. The rows are arranged based on the lncRNAs genomic locations, and each of the rows represents an lncRNA locus. B, The frequency of lncRNAs copy number loss (blue) in thyroid cancer tissues was shown. The rows are arranged based on the lncRNAs genomic locations, and each of the rows represents an lncRNA locus

To date, increasing number of evidence demonstrates that genetic alterations and epigentic are involved in lncRNAs dysregulation in human cancers. To explore whether the genomic copy number variations contribute to lncRNAs differential expression in thyroid cancer, we downloaded the somatic copy number variations data from TCGA. Then, each of those dysregulated lncRNAs loci copy number amplification or deletion frequencies in all thyroid cancer samples were calculated, and *q* value <0.25 was defined as significant. The results of CNV analyses showed that many differentially expressed lncRNAs loci are accompanied with copy number amplification or deletion, such as LINC01354 and LINC01341 have frequency gain, LINC00595 and LINC01519 have frequency loss in thyroid cancer (Figure 2A,B, and Table S2). These findings suggest that lncRNAs dysregulation in human thyroid cancer is partly due to their genomic copy number variations.

### 3.3 Identification of thyroid cancer survival associated lncRNAs

**Figure 3:**
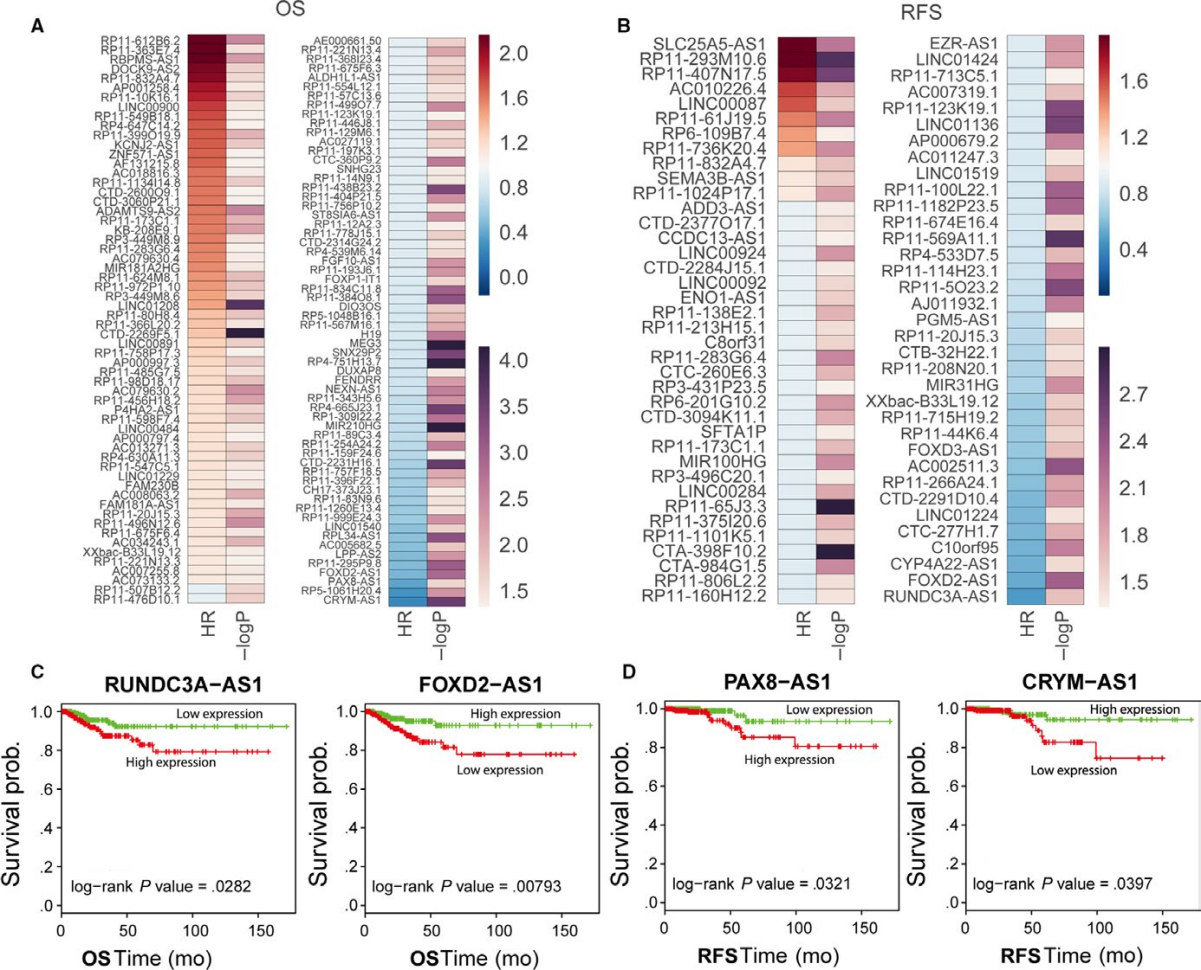
Analysis of thyroid cancer patients’ OS‐ and RFS‐associated lncRNAs. A, B, Heatmaps were drawn to show the log rank *P* value and hazard ratio value of OS and RFS‐associated lncRNAs in thyroid cancer. C, The Kaplan‐Meier curves for thyroid cancer patients’ OS in high‐RUNDC3A‐AS1 or FOXD‐AS1 and low‐RUNDC3A‐AS1 or FOXD‐AS1 groups in the TCGA set. The significant differences between two groups were determined using the two‐sided log‐rank test. D, The Kaplan‐Meier curves for thyroid cancer patients’ RFS in high‐PAX8‐AS1 or CRYM‐AS1and low‐PAX8‐AS1 or CRYM‐AS1groups in the TCGA set

Recently, a growing number of studies have uncovered that a lot of lncRNAs expression levels are related to cancer patients’ survival time, and could be used as independent predictors for cancer patients recurrence‐free and OS time. To determine the relationship between lncRNAs expression and patients’ survival in thyroid cancer, we performed univariable Cox PH regression analysis. The results of univariable Cox PH regression analyses showed that 87 lncRNAs are significantly associated with thyroid cancer patients OS time (log rank *P* < .05), and 63 lncRNAs are significantly related to thyroid cancer patients RFS time (log rank *P* < .05) (Figure 3A, Table S2). Taken RUNDC3A‐AS1, FOXD‐AS1, PAX8‐AS1, and CRYM‐AS1 for example, thyroid cancer patients with higher RUNDC3A‐AS1 or lower FOXD‐AS1 expression levels had shorter OS time, whereas thyroid cancer patients with higher PAX8‐AS1 or lower CRYM‐AS1 expression levels had shorter RFS time (Figure 3B,C). These data suggest that those thyroid‐cancer‐survival‐related lncRNAs may be used as independent predictors for thyroid cancer patients’ survival.

### 3.4 Downregulation of LINC00704 impairs cell proliferation and inhibits cell‐cycle progression in thyroid cancer

**Figure 4:**
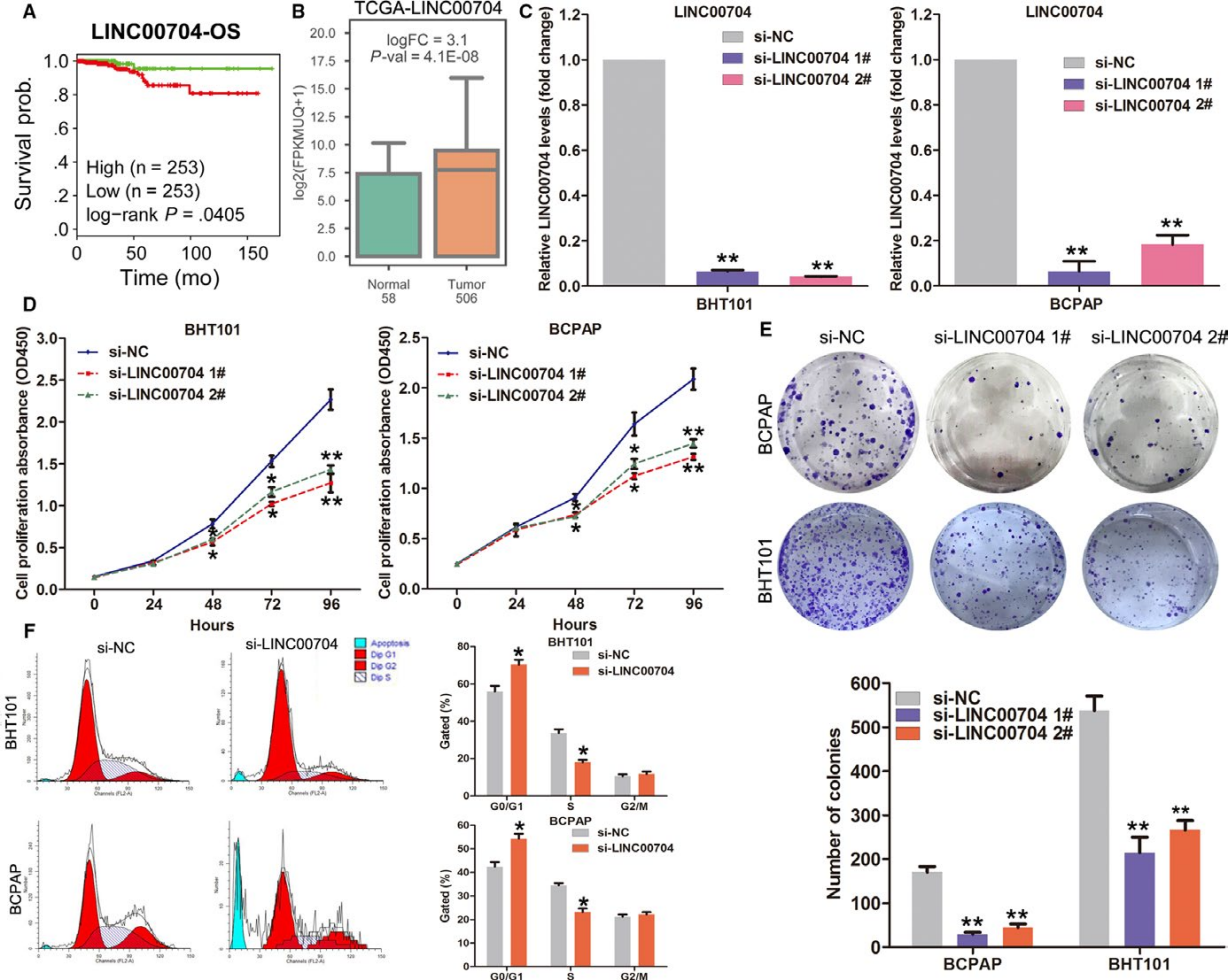
Downregulation of LINC00704 inhibits cell proliferation and cell‐cycle progression in thyroid cancer. A, The fold‐change of LINC00704 expression levels between thyroid cancer tissues and normal tissues in TCGA dataset. B, The Kaplan‐Meier curves for thyroid cancer patients’ OS in high‐LINC00704 and low‐LINC00704 groups in the TCGA dataset. C, The relative expression of LINC00704 in negative control or LINC00704 siRNAs transfected BHT101 and BCPAP cells was detected by qRT‐PCR. D, Growth curves of BHT101 and BCPAP cells after transfection with LINC00704 siRNAs or negative control were determined by CCK8 assays. Values represent the mean ± SD from three independent experiments. E, The colony formation ability of BHT101 and BCPAP cells after transfection with LINC00704 siRNAs or negative control were determined. F, The cell‐cycle progression of BHT101 and BCPAP cells after transfection with LINC00704 siRNAs or negative control was determined using Flow cytometry assays. **P < .01; *P < .05

To validate the analysis results, we chose an lncRNA termed LINC00704 (also known as MANCR) for further investigation. LINC00704 is upregulated in thyroid cancer, and higher LINC00704 expression levels are associated with thyroid cancer patients shorter OS time (Figure 4A,B). Next, we designed specific siRNAs target LINC00704 and transfected them into thyroid cancer cells BHT101 and BCPAP. The results of qPCR analysis showed that LINC00704 expression was significantly decreased in siRNAs transfected cells compared with control cells (Figure 4C). Then, the results of CCK8 assays revealed that knockdown of LINC00704 inhibited BHT101 and BCPAP cells proliferation, and colony formation assays also confirmed that downregulation of LINC00704 impaired BHT101 and BCPAP cells colony formation ability (Figure 4D,E). Moreover, flow cytometry analyses showed that downregulation of LINC00704 could induce BHT101 and BCPAP cells G0/G1 phase arrest. These findings indicate that LINC00704 might play important roles in thyroid cancer development.

### 3.5 Knockdown of LINC00704 induces cell apoptosis and impairs cell invasion in thyroid cancer

**Figure 5:**
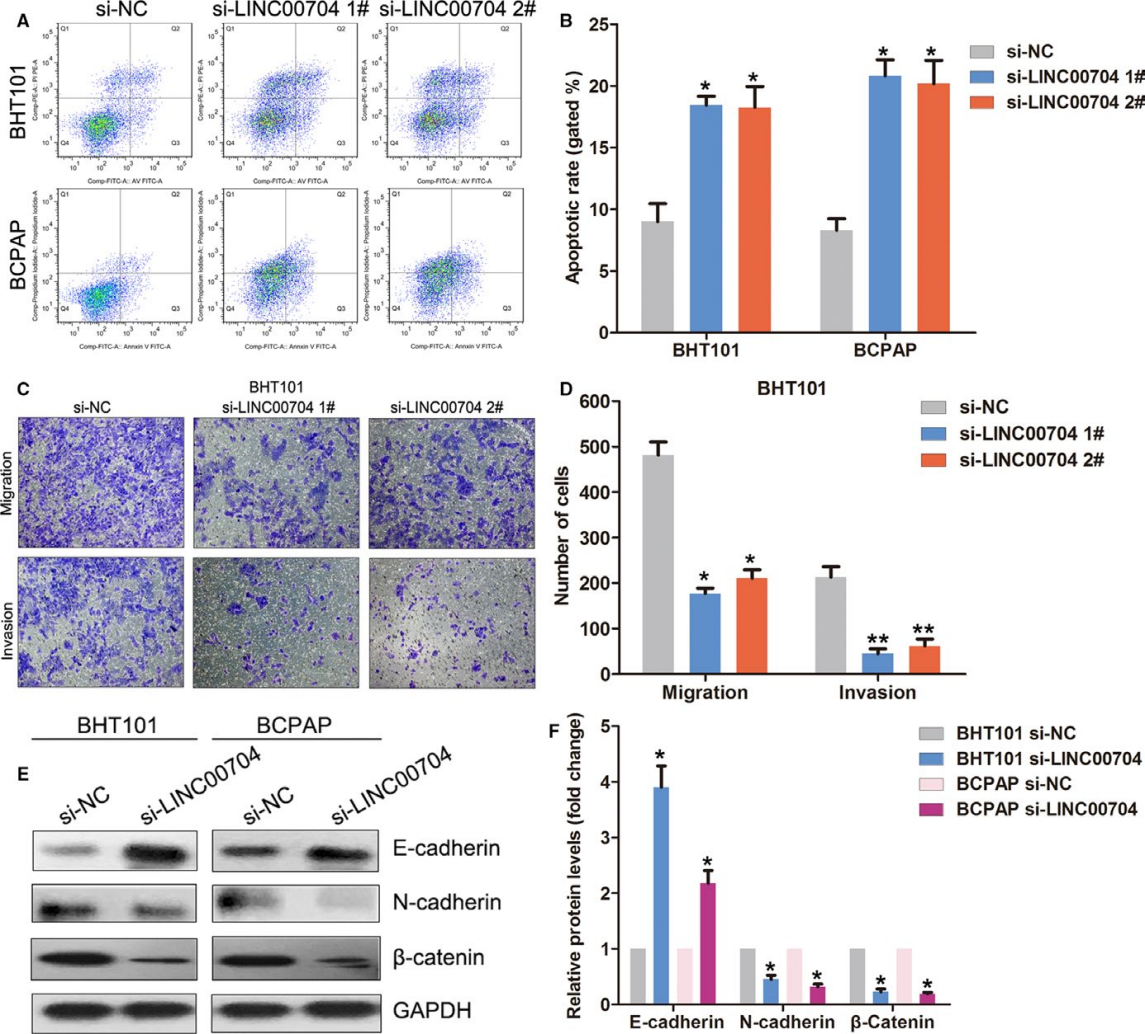
Effect of LINC00704 knockdown on thyroid cancer cells apoptosis and invasion. A, B, The cell apoptotic rate of BHT101 and BCPAP cells after transfection with LINC00704 siRNAs or negative control was determined using Flow cytometry assays. C, D, Transwell assays were performed to evaluate the invasive ability of si‐LINC00704 or si‐NC transfected BHT101 cells. Data represent the mean ± SD from three independent experiments. E, F, The protein levels of E‐cadherin, N‐cadherin and β‐catenin in si‐LINC00704 or si‐NC transfected BHT101 cells were examined by western blot. **P < .01; *P < .05

To further determine whether LINC00704 could affect thyroid cancer cells apoptosis, we performed flow cytometry analyses and the results showed that downregulation of LINC00704 could induce cell apoptosis in BHT101 and BCPAP cells (Figure 5A,B). Meanwhile, the results of transwell assays revealed that knockdown of LINC00704 significantly impaired thyroid cancer cells invasive ability (Figure 5C,D). Recent studies have revealed that epithelial mesenchymal transition plays important roles in tumor cells growth, invasion, and metastasis. To determine whether LINC00704 affects thyroid cancer cells EMT, we detected EMT markers expression levels in LINC00704 downregulated cells. The results of western blot showed that E‐cadherin expression was significantly increased, whereas N‐cadherin and β‐catenin expression was decreased in LINC00704 siRNA transfected BHT101 and BCPAP cells. These data indicate that LINC00704 might involve in thyroid cancer progression through regulating EMT and cell invasion.

## 4 DISCUSSION

Over the past decade, the achievement of whole human genome sequencing has uncovered that the genome noncoding elements were widely transcribed, which yields thousands of long noncoding RNAs.^23^ Accumulating evidence revealed disruptive expression of lncRNAs in various types of human cancers, and these lncRNAs are capable of regulating diverse cellular processes such as tumor cell growth, cell apoptosis, cell invasion and metastasis, and tumor cells sensitivity to chemotherapy drugs.^24^, ^25^ Moreover, a growing number of studies have demonstrated that dysregulated lncRNAs have been identified as critical regulators during the tumorigenesis and cancer progression, and some of these lncRNAs are determined as significant factors for clinical implication. For example, Chen and colleagues reported that over‐expressed LINC001234 is significantly associated with gastric patients poor prognosis and shorter survival time, and promotes cell growth and inhibits apoptosis by acting as miR‐204‐5p sponge and regulating CBFB expression.^26^ In addition, Zhao et al identified downregulated lncRNA FOXD3‐AS1 as an independent prognostic marker for Neuroblastoma patients’ favorable outcome. Moreover, ectopic over‐expression of FOXD3‐AS1 impairs the aggressiveness of neuroblastoma cells through interacting with PARP1 to inhibit the poly(ADP‐ribosyl)ation and activation of CTCF, thereby repressing downstream tumor‐suppressive genes expression.^27^

To date, only a minority of lncRNAs’ clinical relevance, biologic function, and underlying mechanisms have been characterized in thyroid cancer. For example, NEAT1_2 expression was significantly upregulated in papillary thyroid cancer tissues, and increased NEAT1_2 expression was positively related to patients TNM stage and tumor size; downregulation of NEAT1_2 resulted in significant inhibition of cell growth and metastasis by acting as a competing endogenous RNA for miR‐106b‐5p to regulate ATAD2 expression.^28^ In addition, lncRNA HCP5 is overexpressed in papillary thyroid cancer and promotes proliferation, invasiveness, and angiogenic ability of papillary thyroid cancer cells through functioning as a sponge for miR‐22‐3p, miR‐186‐5p, and miR‐216a‐5p and antagonizing their repression of ST6GAL2.^29^ In this study, we performed a comprehensively analysis of lncRNAs profile in thyroid cancer and identified thousands of novel dysregulated lncRNAs in thyroid cancer tissues, such as upregulated UNC5B‐AS1 and LINC01510, and downregulated EPHA5‐AS1 and LINC01384. Moreover, some of those dysregulated lncRNAs genomic loci contains copy number amplification or deletion, such as LINC01354, LINC01341, LINC00595, and LINC01519, suggesting that genomic alterations might involve in part of these lncRNAs dysregulation in thyroid cancer. Additionally, we identified a few of lncRNAs expression levels are significantly associated with thyroid cancer patients OS or RFS time, such as RUNDC3A‐AS1, FOXD‐AS1, PAX8‐AS1, and CRYM‐AS1, suggesting that lncRNAs could be useful prediction factors for thyroid cancer patients survival.

To validate our analyses results, we chose an upregulated lncRNA LINC00704 which was also known as MANCR and investigated its function in thyroid cancer. Increased LINC00704 expression levels are associated with shorter OS time in thyroid cancer patients, and knockdown of LINC00704 significantly impaired proliferation and colony formation capacity of thyroid cancer cells. Moreover, we found that downregulation of LINC00704 could induce cell G1/G0 phase arrest and cell apoptosis, and inhibited cell invasive ability in thyroid cancer. In addition to our findings, LINC00704 was found to be upregulated in breast cancer specimens and cells. Depletion of MANCR significantly inhibits triple‐negative breast cancer cells proliferation with concomitant increases in DNA damage and increase the incidences of cytokinesis and cell apoptosis through affecting the expression of >2000 genes that enriched in cell‐cycle regulation pathway.^30^ In this study, we found that LINC00704 could affect epithelial mesenchymal transition (EMT) process by regulating E‐cadherin, N‐cadherin, and β‐catenin expression in thyroid cancer. EMT is a biologic process by which epithelial cells lose their polarity and gain migratory and invasive properties to transition into a mesenchymal phenotype.^31^ EMT has been determined to be essential for numerous developmental processes, and increasing evidence reveals that EMT also occurs in the initiation of tumor cells metastasis during cancer progression.^32^ These findings suggest that LINC00704 might play a critical role in thyroid cancer tumorigenesis and progression, which partly depends on regulation of cell EMT.

In summary, findings in the present study revealed that thousands of lncRNAs were differently expressed in thyroid cancer tissues compared with their parental normal or non‐tumor tissues. A small portion of dysregulated lncRNAs are significantly associated with thyroid cancer patients OS and RFS time, which might play important roles in thyroid cancer development and progression. Our findings highlight the key roles of lncRNAs in thyroid cancer, and may provide a useful list of lncRNA candidates as potential diagnostic markers and targets for thyroid cancer therapy. However, there are also a few limitations in the present study, for example, only candidate LINC00704 was validated and its underlying molecular mechanism remains unclear, which is needed to be further investigated in the future work.

## CONFLICTS OF INTEREST

No potential conflicts of interest were disclosed.
